# Acute Respiratory Infection Hubs: A Service Model with Potential to Optimise Infection Management

**DOI:** 10.3390/antibiotics12050819

**Published:** 2023-04-27

**Authors:** Sarah Jawad, Anna Buckingham, Charlotte Richardson, Aoife Molloy, Bola Owolabi, Matt Inada-Kim

**Affiliations:** 1 Department of Infection Sciences, King’s College Hospital, Denmark Hill, London SE5 9RS, UK; 2 NHS England, Wellington House, London SE1 8UG, UK; 3 Infectious Diseases Department, Royal Free Hospital NHS Trust, London NW3 2QG, UK; 4 Institute of Applied Health Research, College of Medicine and Dentistry, University of Birmingham, Birmingham B15 2SQ, UK; 5 Clinical & Experimental Sciences, University of Southampton, Southampton SO17 1BJ, UK; 6 Wessex Academic Health Science Network, Chilworth, Southampton SO16 7NP, UK; 7 Hampshire Hospitals NHS Foundation Trust, Hampshire, UK

**Keywords:** respiratory, infection, hub, inequalities, stewardship, interface

## Abstract

Patients with acute respiratory infections (ARI)—including those with upper and lower respiratory infections from both bacterial and viral pathogens—are one of the most common reasons for acute deterioration, with large numbers of potentially avoidable hospital admissions. The acute respiratory infection hubs model was developed to improve healthcare access and quality of care for these patients. This article outlines the implementation of this model and its potential impacts in a number of areas. Firstly, by improving healthcare access for patients with respiratory infections by increasing the capacity for assessment in community and non-emergency department settings and also by providing flexible response to surges in demand and reducing primary and secondary care demand. Secondly, by optimising infection management (including the use of point-of-care diagnostics and standardised best practise guidance to improve appropriate antimicrobial usage) and reducing nosocomial transmission by cohorting those with suspected ARI away from those with non-infective presentations. Thirdly, by addressing healthcare inequalities; in areas of greatest deprivation, acute respiratory infection is strongly linked with increased emergency department attendance. Fourthly, by reducing the National Health Service’s (NHS) carbon footprint. Finally, by providing a wonderful opportunity to gather community infection management data to enable large-scale evaluation and research.

## 1. Introduction

The COVID-19 pandemic resulted in rapid innovations in service delivery in response to the extraordinary demand for acute healthcare services. Acute respiratory infections from a range of pathogens remain a significant source of pressure in the aftermath of the pandemic and require a sustainable, efficient, patient-centred, and effective care model. The acute respiratory infection (ARI) hub model seeks to address this pressure and was rapidly implemented across England this year in response to the recent large surge in demand.

During the 2022/23 winter season, the ARI burden was 2.5–3 times greater than baseline activity compared to previous years [1], which contributed to the NHS facing unprecedented challenges in emergency care access and capacity. The burden of respiratory infections from specific and sometimes overlapping pathogens was especially high, including severe acute respiratory syndrome coronavirus-2 (SARS-CoV-2), influenza, respiratory syncytial virus (RSV), and group A streptococcus. UK Health Security Agency (UKHSA) surveillance data demonstrated high numbers of cases and hospitalisations from these infections [1]. ARI forms a significant part of both the primary and secondary care burden, with 10–55 ARI non-admitted patients per emergency department (ED) per day in England [2]. We define acute respiratory infections as those presenting with suspected ARIs, including upper and lower respiratory tract infections (including pneumonia) from viral (e.g., COVID-19, influenza, RSV) and bacterial pathogens within 21 days of symptom onset.

ARI hubs provide suspected ARI patients with urgent, same-day appointments. They provide face-to-face assessment by an appropriate clinician in a non-hospital setting, with access to appropriate rapid diagnostics and treatments. The hubs are designed to meet local needs and integrate into local care systems.

ARI hubs are a novel integrated care approach with the potential to improve patient outcomes and address service pressures by avoiding unnecessary hospital attendance, facilitating effective antimicrobial stewardship, and tackling health inequalities with improved access to care. They also seek to empower and educate patients, whilst also potentially reducing carbon dioxide (CO_2_) emissions.

## 2. Learning Post-COVID-19

COVID-19 led to unprecedented pressures on healthcare environments globally, requiring adapted or newly designed pathways to facilitate safe admission avoidance and/or early discharge from hospital for ongoing community care. However, access to same-day care and prompt assessment of clinical conditions in the community were challenging. The ARI hub model offers an innovative solution to this challenge. Across the NHS, virtual wards have become widespread as a safe method to facilitate discharge and monitor complex patients who may not require hospital admission [3]. Most of these models are led by secondary care and involve a range of techniques, including online platforms, phone calls, and use of wearable technology [4]. The use of technology in remote monitoring was successfully trialled in novel services such as the COVID-19 oximetry @home model that was rapidly implemented across the whole of the NHS [5]. The empowerment and education of patients were identified as key measures of success, leading to a reduction in both the need for hospitalisation and mortality rates [6]. The pandemic also increased our (both patients and clinicians) familiarity with the use of point-of-care testing to diagnose and manage infections, and the ARI hubs represent an opportunity to standardise this approach and evaluate its usage.

Winter months are typically associated with a surge in respiratory infections; however, there has been a shift in previously recognised patterns due to the effects of multiple lockdowns, leading to a persistently high case incidence of ARIs [7]. There has also been a noted overlap of respiratory infections over the winter of 2022/23, with a rise in group A streptococcus cases alongside high influenza and RSV rates. This has inevitably led to increased numbers of patients requiring assessment for superimposed bacterial infections and requiring early antimicrobial management [8]. Reduced antimicrobial availability and unprecedented pressures in both primary and secondary care settings expose the need for new models of care, building on the rapid innovation that resulted from the COVID-19 pandemic. In England, patient satisfaction with primary and secondary care has dropped, mainly due to problems with access [9], highlighting the need for urgent improvements in accessible and coordinated healthcare in the community.

## 3. ARI Hub Service Model

The urgent care pathway in primary and emergency care is under significant strain, and there is a clear need for a flexible and inclusive management solution for undifferentiated acute respiratory infections (ARI) in the community. ARI is one of the most common reasons for consulting in general practise or attending emergency departments in the UK [10,11]. The majority of patients that attend emergency departments with ARI symptoms are not admitted, which suggests they could be safely reviewed and treated in the community. The level of ARI-related activity in 2022 has continued at high levels, with a peak of over 70,000 ARI attendances per week at emergency departments in England in late December 2022, double the peak of 2021 [12]. Over the summer months, there is usually a significant reduction in ARI activity (in both primary and secondary care); however, this has not occurred over the last two years (2020–2022). This unseasonal activity is set to continue due to an ageing population and further fluctuations in viral infections.

**Figure 1:**
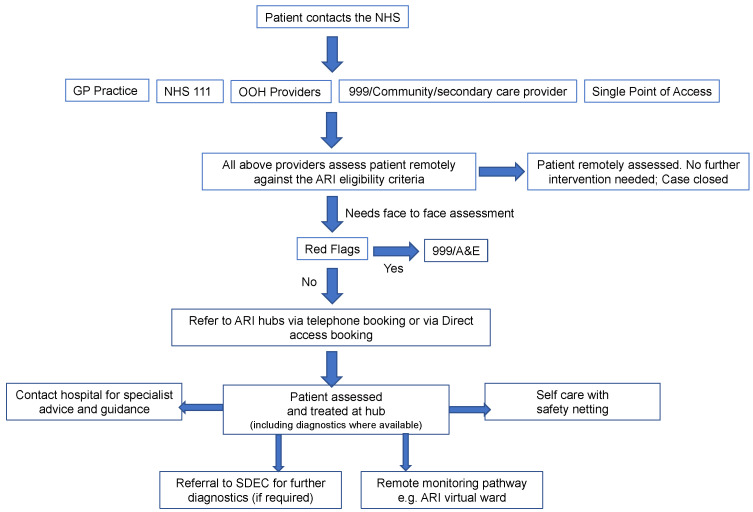
Illustrative example of an ARI hub pathway. Abbreviations: out of hours (OOH), same- day emergency care (SDEC), NHS England: Combined adult and paediatric acute respiratory infection (ARI) hubs (previously RCAS hubs), October 2022 [14].

The Fuller stocktake [13] published in May 2022 examined primary care in England and highlighted the need for “streamlined access to urgent, same-day care and advice from an expanded multi-disciplinary team”. The ARI hubs fit this model perfectly, providing an integrated pathway from each patient’s first contact with healthcare services to diagnostic tests, treatment, monitoring, and recovery. The ARI hubs facilitate this model through standardised and prompt triage to the appropriate level of care, urgent same-day review, integrated community and acute care teams, and access to point-of-care diagnostics and treatments. The combined adult and paediatric respiratory infection (ARI) hub guidance (published October 2022) developed by NHS England, an executive non-departmental public body that leads the NHS in England, outlines how local systems can establish care models to deliver increased capacity and access to same-day urgent review (Figure 1) [14]. This is delivered in a non-hospital setting by clinicians, and is integrated into the local primary care, community care, hospital, and virtual ward programmes. This initiative was provided with support for winter 2022–23, allowing systems to increase capacity for the management of acute respiratory infections. NICE are rapidly developing clinical guidance on the initial assessment and management of undifferentiated patients presenting with acute respiratory infection symptoms to support the ARI hubs [15].

ARI hubs allow systems to create a flexible response to surges in acute respiratory infections (e.g., COVID-19, influenza, RSV infections, and group A strep). By improving access for these patients in non-ED and general practise (GP) settings, a significant proportion of ED attendances might be avoided and primary care capacity released to focus on primary prevention and long-term condition management. ARI hubs may also reduce nosocomial transmission of COVID-19 and other infectious conditions by cohorting symptomatic patients. The ARI hubs provide an opportunity to bring diagnostics closer to patients through point-of-care testing for COVID-19, influenza, and blood tests, including C-reactive protein (CRP). These can lead to improved antimicrobial stewardship and reduce healthcare inequalities by ensuring more equitable access to appropriate antiviral treatments [16].

Acute respiratory infection hubs are designed to provide high quality care for those with ARI symptoms who require acute assessment but do not necessarily require hospital admission. They offer same-day face-to-face appointments with a multidisciplinary team including GPs, advanced nurse practitioners, virtual hospital specialists, and other healthcare professionals, providing a more personalised care approach. NHS England has supported the rapid expansion and roll-out of ARI hubs across the country in winter 2022/23, allowing local systems to build on existing infrastructure and manage the increased demand. These can be paediatric-focused, adult-focused, or both and are developed by regions to address local needs.

NHS England provided significant funding for ARI hubs and asked regions to focus on areas of deprivation with poor healthcare access. They are mainly located in community settings and operate at hours of peak demand, from afternoons to late evenings on weekdays, and full days on weekends. By building links and pathways with emergency departments, they can safely divert patients away from the hospital when pressures increase.

## 4. Antimicrobial Stewardship

Antimicrobial stewardship (AMS) and antimicrobial resistance (AMR) are significant global healthcare challenges, continuing to be high priorities within national and international policy agendas [17]. Concerns that the pandemic compromised AMS have meant enhanced surveillance of antibiotic prescribing and usage. ARI hubs represent an opportunity to collect data and for collaboration with microbiology and infection specialist services to improve antimicrobial stewardship in this interface setting. Rapid and robust antimicrobial susceptibility testing is ideal but not always available in real-time, so empirical management strategies and guidelines have been designed at local and national levels. National clinical guidelines are used, e.g., FeverPAIN and Centor clinical prediction scores for patients presenting with sore throats in primary care, as recommended by the National Institute for Health and Care Excellence [18], to promote effective stewardship and reduce inappropriate antibiotic prescription, supported by clinical microbiology specialist services. In addition, rapid point-of-care (POC) diagnostics in community settings can help reduce the misuse of antibiotics, e.g., capillary blood sampling for measuring CRP and procalcitonin [19]. The hubs also offer an opportunity for effective team-based decisions, improvement, and training. Multi-disciplinary approaches with effective collaboration with personnel, such as community pharmacists, present an opportunity to enhance diagnostics and decision-making, and therefore, stewardship [20].

In summary, the ARI hubs offer a combination of same-day access to face-to-face clinical assessment, best practise clinical pathways, and the measurement of outcomes, which are central to enhancing AMS and safely curbing antibiotic prescribing. The model presents an opportunity to ally point-of-care diagnostics with presentation and outcomes, while also using demographics to capture data and create a mass population-based model of identifying key presenting factors that indicate when antibiotics are appropriate and what predicts the best outcomes for patients. Further research needs to be undertaken in this area, and ARI hubs offer a huge opportunity to gather information on demographics, outcomes, use of diagnostic tests, frequency and choice of antibiotic prescribing, and patient satisfaction.

## 5. Inequalities and Respiratory Infections

The NHS Long Term Plan 2019 [21] and Operational Planning Guidance 2023/24 [22] commit to stronger action on health inequalities, defined as unfair and avoidable differences in health across the population and between different groups within society. Restoring NHS services inclusively is one of five key priorities, and in 2021 the National Healthcare Inequalities Improvement Programme (HiQiP) was established to work with programmes across NHS England, as well as with partners in the wider system, patients, and communities, to deliver exceptional quality healthcare for all, ensuring equitable access, excellent experience and optimal outcomes.

The Core20PLUS5 approach for adults [23] and the Core20PLUS5 approach for children and young people (CYP) [24] were developed by NHS England HiQiP to inform action to reduce healthcare inequalities at both the national and system levels. The approach defines a target population of the most deprived 20% of the national population, as identified by the national Index of Multiple Deprivation [25] and locally identified PLUS groups at greater risk of experiencing inequalities. These may include people from ethnic minority communities; people living with a learning disability and autistic people; people living with multiple long-term health conditions; other groups that share protected characteristics, as defined by the Equality Act 2010; people from coastal communities (where there may be small areas of high deprivation hidden amongst relative affluence) and inclusion health groups, such as vulnerable migrants; people experiencing homelessness; and people with drug and alcohol dependence. The approach also centres around ‘5’ key clinical areas of focus that require accelerated improvement. These include asthma in children and young people and chronic respiratory disease in adults, representing one of the biggest contributors to the gap in life expectancy between the most- and least-deprived populations, with a target of reducing infective exacerbations and emergency hospital admissions.

Multiple factors contribute to inequalities in the burden of respiratory disease, including differences in smoking rates, exposure to outdoor air pollution, poor housing, and occupational exposure [26]. Respiratory infections disproportionately affect those in the most deprived groups, with higher inpatient admission rates for respiratory infections in deprived populations [27]. There is an association between deprivation and worse clinical outcomes in patients admitted with community-acquired pneumonia [28], and the rate of deaths involving COVID-19 in the most deprived areas in England is double that of the least deprived [29]. Acute exacerbations of chronic obstructive pulmonary disease (COPD) account for roughly 1 in 8 emergency hospital admissions in England [30], with the rate for those in the most deprived areas being more than triple that in the least deprived [31]. Smoking is a known risk factor for acute respiratory infections [32], and tobacco use is strongly linked to inequality, with four times higher smoking prevalence in the most deprived decile compared to the least deprived [33], and higher rates in those with a mental health condition [34].

Inequalities also influence the place of care, with a British Red Cross report showing high-intensity use of EDs to be closely associated with deprivation, pre-existing mental health conditions, housing insecurity, and social isolation [35]. The major reasons for acute deterioration in patients with chronic respiratory conditions are acute respiratory infections, and a 5-year study of asthma care in Wales demonstrated that, compared to the least deprived patients, the most deprived patients had fewer attendances for routine review and higher asthma-related emergency admissions and inpatient stays, with a higher risk of asthma-related death [36]. For patients with COPD, deprivation is linked to lower secondary care outpatient appointment attendance, lower participation in exercise rehabilitation, and increased emergency care use [37]. These findings suggest inequalities in the prevention and optimisation of chronic respiratory disease, which may lead to increased emergency presentations.

Global inequalities in access to antibiotics, population behaviour, and antimicrobial stewardship contribute to a variation in levels of antimicrobial resistance [38]. In Europe, there is increased antibiotic resistance among refugees and asylum seekers [39], and a study of developed European countries, including the UK, demonstrated that income inequality is positively correlated with antimicrobial resistance in common bacterial infections [40]. Within the UK, there is evidence for differential prescribing of antibiotics, with higher levels of antibiotic prescribing and antimicrobial resistance in the most deprived areas [41]. A recent report on prescribing patterns in England showed inequalities in prescription exemption uptake, burden of medications at earlier ages, and polypharmacy between deprived and non-deprived communities. This report suggests that inequalities in prescribing experienced by people in deprived communities may be widespread [42].

ARI hubs represent an opportunity to tackle inequalities by ensuring equitable healthcare access to high-quality care, best-evidenced pathways of care, and point-of-care diagnostic tests, leading to optimal outcomes for all. NHS England has asked for the prioritisation of ARI hubs in areas of deprivation to avoid exacerbating healthcare inequalities. Co-design of pathways with patients and communities is essential to promoting equitable access for underserved populations. By utilising the opportunity of the patient interaction using a ‘Make Every Contact Count’ (MECC) approach [43], ARI hubs could also support the implementation of Core20PLUS5 interventions, such as promoting vaccination uptake in chronic respiratory disease, hypertension case-finding in adults, and inhaler optimisation for asthma in adults, children, and young people.

## 6. Patient Education and Behaviour Change

Patient education, and by extension, public health and education, are at the core of the patient-centred approach used in the ARI hubs. Influencing behaviour change seeks to empower patients with the knowledge and reassurance needed to both identify predictors of severe respiratory illness requiring escalation and promote confidence in decisions that do not involve antibiotic prescribing. Education and communication with patients and carers are key; this is particularly relevant in the context of ARIs, whereby a large proportion of patients presenting to primary care are either recurrent attendees with chronic illnesses or children. Effective communication, signposting of resources, and education of parents have consequent effects on the use of emergency services, antimicrobial stewardship, and patient satisfaction. Offering appropriate patient information on self-care and risk stratification, as in the Healthier Together campaign [44], can help reduce patient morbidity, particularly in those with underlying respiratory conditions who are at a high risk of presenting with acute deteriorations, particularly in the winter months. ARI hubs provide a safe and accessible bridging service for these cohorts of patients and an opportunity to receive education and information in a dedicated and specialised environment.

## 7. ARI Hubs and the Greener NHS

In 2020, NHS England committed to becoming the world’s first net-zero national health system by 2040 for the emissions it has the ability to control, and by 2045 for the emissions it has the ability to influence [45]. The ambition to achieve net zero carbon emissions is shared by all four UK health services. In 2019, the carbon footprint of the NHS in England was calculated to be around 25 megatonnes of CO_2_ equivalent. Acute care represents the most carbon-intensive care pathway, with 125 kg of CO_2_ equivalent per day for hospitalised patients versus 66 kg CO_2_ equivalent per general practise visit [46]. Travel to and from NHS sites by patients and visitors and staff commuting represents 10% of these emissions.

There are other associations between health inequalities, sustainability, and poor respiratory health. Metered-dose inhalers used in the management of exacerbations of chronic respiratory disease are also a source of greenhouse gas emissions, and their use is higher in the Core20 population, which is prescribed more COPD medications than the non-Core20 population [42]. There are inequalities in how air pollution affects populations in terms of exposure to air pollutants and susceptibility to their health effects. Children, older people, and people with chronic health problems are the most vulnerable to short-term episodes of high air pollution levels. People in low-income communities and some minority ethnic communities are more likely to be affected by air pollution, and the most deprived communities in England tend to have the highest levels of air pollution. An estimated 26,000–38,000 deaths occur every year from poor air quality [47].

New service models that optimize the location of care based on clinical need ensure efficient access to the most appropriate services, which may be closer to their homes. ARI hubs providing ‘out-of-hospital’ care may reduce emissions by helping to avoid unnecessary hospital visits and admissions [45].

## 8. ARI Hubs and Research

ARI hubs provide an opportunity for research in a novel clinical setting to investigate the impact of this model across the health and care system on avoidable admissions, optimal outcomes (e.g., treatment success, deaths, intensive care admissions, length of stay, costs of care, readmissions), antimicrobial stewardship, healthcare inequalities, and patient satisfaction. Embedding service evaluation is essential to identifying potential benefits and best practises that may be replicated and built on in future service development.

## 9. Conclusions

The COVID-19 pandemic accelerated learning and innovation in the delivery of services, the development of treatment pathways, and the use of novel therapeutics. The use of technology for virtual patient monitoring, risk-stratifying tools, and improving patient education in identifying early clinical deterioration reduced healthcare demand at a time of great strain across the NHS. Following on from this, ARI hubs represent an opportunity to optimise infection management and patient experience in the context of the current challenges facing the NHS and the high burden of respiratory pathogens. They also build capacity for acute services to rapidly respond to future surges in demand.

We must embrace the opportunity provided by the expansion of the ARI hub model to tackle healthcare inequalities by promoting equitable access to high-quality care, health education, appropriate antibiotic prescribing, and delivering positive patient experiences and outcomes. Co-design with target populations, considering the location of services, and focusing on underserved communities will help narrow the gap in healthcare inequalities. ARI hubs located in proximity to the communities they serve also support the NHS Net Zero agenda, with the potential to reduce CO_2_ emissions via a reduction in unnecessary hospital visits and admissions.

Finally, ARI hubs represent an opportunity for collaboration with microbiology and infection specialists to promote antimicrobial stewardship (and minimise AMR) and truly optimise infection management. More research is needed in this area to develop appropriate and evidence-based guidelines for this interface medicine setting, but at a time of considerable NHS pressure, this innovative model presents an approach that prioritises optimal clinical management, patient-centred care, and scope for learning, development, and research.

## Data Availability

The NHS England data referenced in this study is available on request from the corresponding author. The data is not publicly available due to its being, as of yet, unpublished.
